# Use of Different Synbiotic Strategies to Improve Gut Health in Dogs

**DOI:** 10.3390/ani14233366

**Published:** 2024-11-22

**Authors:** Miquel Montserrat-Malagarriga, Lorena Castillejos, Anna Salas-Mani, Celina Torre, Susana María Martín-Orúe

**Affiliations:** 1 Nutrition and Welfare Service, Department of Animal and Food Science, Universitat Autònoma de Barcelona, Cerdanyola del Vallès, 08193 Barcelona, Spainsusana.martin@uab.cat (S.M.M.-O.); 2 Affinity Petcare, Hospitalet de Llobregat, 08902 Barcelona, Spain; asalas@affinity-petcare.com (A.S.-M.); ctorre@affinity-petcare.com (C.T.)

**Keywords:** synbiotic, dietary fiber, prebiotic, probiotic, dried plasma, immunity, dog, microbiota

## Abstract

This study evaluated the effects of two synbiotic strategies on the intestinal microbiota and immune response in Beagle dogs. Twelve dogs were subjected to a crossover design with three diets: a control diet (CON), a diet supplemented with fiber and *B. velezensis* DSM 15544 (SYN), and the SYN diet with added porcine plasma (SYN+). Over three periods of seven weeks, fecal samples were analyzed for digestibility, short-chain fatty acids (SCFA), fecal markers, and microbiome composition, while blood samples were assessed for biochemical parameters, leucocytic counts including CD4/CD8 lymphocyte populations, and phagocytic activity. Both SYN and SYN+ diets increased the fecal volume without affecting the consistency and slightly reduced the organic matter and energy digestibility, while increasing SCFA concentrations and reducing branched-chain fatty acids. A microbiome analysis revealed no changes in the alpha diversity, but significant shifts in the beta diversity, with increases in beneficial taxa such as *Faecalibacterium prausnitzii* and reductions in potentially harmful bacteria like *Prevotella copri*. Immune response indicators showed increased fecal IgA and higher blood leukocyte counts, particularly lymphocytes and neutrophils, in the SYN diet group. Overall, both synbiotic strategies positively modulated the microbiota and immune response, though the addition of porcine plasma did not confer additional benefits.

## Simple Summary

This study examined two different dietary strategies designed to improve gut health and immune function in dogs. Synbiotic combinations of specific probiotic strains with dietary fiber are well-known for supporting gut health. Additionally, these beneficial effects may be enhanced by supplementing these diets with functional ingredients such as porcine plasma, which could offer additional benefits. In this study, researchers tested three diets: a regular diet, a diet enhanced with fiber and a specific probiotic, and the same enhanced diet with added porcine plasma. Both experimental diets improved gut bacteria and maintained good stool quality, although they slightly reduced the digestion of some nutrients. These diets also boosted the dogs’ immune responses by increasing certain white blood cells or immune markers in the stool. Overall, this study suggests that both synbiotic strategies tested can enhance gut health and immune function in dogs, potentially leading to better overall health and well-being, even though the addition of porcine plasma did not produce distinctive effects in this study.

## 1. Introduction

The concept of a functional ingredient has been evolving and adapting over the years [1]. Although definitions vary depending on the institution and author, the Food and Agriculture Organization (FAO) defines functional food as “a foodstuff that provides health benefits beyond basic nutrition, demonstrating specific health or medical benefits, including the prevention and treatment of disease” [2]. Other definitions have been proposed, but they consistently emphasize that functional foods offer health benefits that go beyond basic nutritional value [3,4]. A wide range of ingredients could fall under these definitions, but some of the most significant functional ingredient groups include prebiotics, probiotics, or the combination of both, known as synbiotics.

In essence, prebiotics are considered “a substrate that is selectively utilized by host microorganisms, conferring health benefits” [5]. Among the most studied prebiotic ingredients are different non-digestible oligosaccharides like fructooligosaccharides [6], but some fibrous ingredients, which are a source of multiple fermentable compounds, can also exhibit prebiotic effects. For example, beet pulp could serve as a source of pectic polysaccharides that have been described to have great fermentation characteristics [7], and notable prebiotic effects in dogs [8].

Probiotics are defined as “live microorganisms which, when administered in adequate amounts, confer a health benefit on the host” [9]. Many probiotic strains have been tested in dogs with positive effects. Most of the studied strains belong to the *Lactobacillus* or *Bifidobacterium* genera [10,11,12], but other genera like *Enterococcus* and *Bacillus* have also demonstrated beneficial effects [11,12,13]. Particularly, the probiotic *Bacillus velezensis* DSM 15544 (previously known as *Bacillus subtilis* C-3102) has demonstrated beneficial effects on dogs’ fecal odor and quality, microbiota communities, and nutrient digestibility [14,15,16,17]. This strain appears to be a useful probiotic for pet food, considering its stability in feed when handled in its sporulated form.

Synergistic synbiotics are intended to improve the survival of the probiotic as well as its implantation in the gastrointestinal tract [18]. Prebiotics are supposed to provide additional support for probiotic growth and resistance, especially in the upper gastrointestinal tract, where fewer nutrients are available, and the conditions are less favorable. Therefore, synbiotics should be able to enhance the isolated beneficial effects of each of their components [19]. There have been a few studies in dogs testing different synbiotic combinations. Fructooligosaccharides are the most common prebiotic source used in synbiotics as they are well fermented by most probiotics [20]. On the other hand, a range of *Lactobacillus*, *Enterococcus*, and *Bifidobacterium* are normally used as probiotics [12]. The effects commonly reported with synbiotic use include increases in the microbiota diversity [12,21], positive shifts in microbial populations, and a reduction in the incidence of diarrhea [21,22]. However, results can vary greatly and highly depend on the compounds being tested, doses, and forms of administration.

Besides “biotics”, other ingredients could also be categorized as functional. Blood plasma is one such ingredient. Normally administered in the form of Spray-Dried Plasma (SDP), it is mainly composed of serum proteins, primarily albumin and immunoglobulins [23]. SDP has been garnering interest in recent years. It has been used as a functional ingredient in animal production, showing benefits in animal performance by improving intestinal health and positively modulating the immune system and microbiota [23,24,25,26]. There is limited evidence of the effects of SDP in dogs, even though it is commonly used in wet pet food products for its texture-enhancing and gelling properties [27]. Studies in dogs have shown that the addition of SDP does not affect the food intake, can increase nutrient digestibility, and decrease the fecal output [28,29]. Additionally, high doses of SDP have been reported to increase circulating levels of leukocytes in dogs [29]. Interestingly, dietary IgG present in SDP has been shown to resist the digestion process in dogs [30] and decrease the adherence of enteropathogens in an in vitro canine gut model [31].

Therefore, the main objective of this study was to explore the potential of a synbiotic strategy combining diverse fibers and prebiotic sources (including inulin, wheat byproducts, pea fiber, and sugar beet pulp) and a probiotic strain (*B. velezensis* DSM15544) to improve dogs’ intestinal health and immunity. Moreover, this study also aimed to test possible further benefits of additional supplementation with SDP.

## 2. Materials and Methods

The Animal Protocol Review Committee of Universitat Autònoma de Barcelona (UAB) approved the experimental procedure, following the European Union Guidelines for the ethical care and handling of animals under experimental conditions with Register Number CEEAH: 3010; DMAH: 10166. A total of 12 healthy neutered adult Beagle dogs (6 males of 14.8 ± 1.98 kg and 6 females of 13.0 ± 1.57 kg, 4–5 years old) were used for this study. The dogs were divided into three groups of four based on sex and body weight, with each group consisting of two males and two females and an approximately equivalent average body weight (13.6 kg, 13.2 kg, and 13.0 kg at the start of the study, respectively). The control group also used a separated space for daily walks to avoid possible exposure to the probiotic strain through residual fecal material. The animals were housed in a shared space with their group and were separated into individual kennels only during the sampling week to facilitate complete fecal collection. They had daily supervised walks in an enclosed outdoor area. Indoors, temperature was controlled between 18 and 25 °C, but there was no automated lighting system. Each group had access to four open kennels and a common area, with one water bowl securely attached to the wall in each kennel, ensuring a constant supply of fresh, clean water. The kennels were also equipped with an elevated platform, providing animals a place to rest or hide underneath.

### 2.1. Diets

Three experimental dry extruded complete diets were tested in this study (manufactured by Affinity Petcare SA, Hospitalet de Llobregat, Barcelona, Spain). First, a control diet low in fiber (CON) based on chicken, corn, whole wheat, broken rice, poultry fat, hydrolyzed animal protein, fish oil, minerals, and vitamins, and then the same diet but enriched with several sources of dietary fiber (beet pulp 3.00%, pea fiber 1.76%, wheat by-products 1.72%, and 0.8% of inulin) and a probiotic (Calsporin^®^ (Calpis America, Peachtree City, GA, USA)), 1 × 10^6^ CFU/g diet, *B. velezensis* DSM 15544) (SYN). Finally, the last diet consisted of the SYN diet supplemented with 0.4% Spray-Dried Plasma (SYN+). Spray-dried plasma was a protein product (73% crude protein) composed of pure porcine albumin and globulin proteins, with preserved biological characteristics. The total immunoglobulin content was estimated at 200 g/kg. The analyzed nutrient concentrations of the three diets are presented in Table 1. The individual amount of food was offered in a bowl once a day in the morning and adjusted to maintain their body weight. There were no instances of food refusal, which was recorded daily during the study period, and the animals did not have access to any other food sources within the kennels or common areas.

### 2.2. Experimental Design

Diets were tested in a crossover design comprising three periods, each lasting 7 weeks (12 replicates per treatment), resulting in a total study duration of 21 weeks. Each group of dogs switched to a different diet during each of the three study periods. Prior to the initial period, animals were fed an acclimation diet similar to the control diet for 4 weeks. Each period included a first transition week, where animals were gradually introduced to the new diet over 3 days, followed by 5 weeks of adaptation and a seventh week designated for sampling.

During the sampling week, total feces were collected for 6 days for nutrient digestibility analysis. Thorough controls were implemented during daytime (from 8:00 a.m. to 5:00 p.m.) to collect feces and guarantee its integrity. Before and after feces collection, a marker (FeO^3^) was added to the food to ensure that only feces that belonged to the digestibility trial were collected. Additionally, less than 15 g of fresh feces (collected within 30 min of defecation) were gathered during the sampling week and divided into five aliquots for the determination of microbiota, short-chain fatty acids, IgA, calprotectin, and NH_3_, with sample weights of 1 g, 3 g, 1 g, 1 g, and 2 g, respectively. The NH_3_ sample was placed in a tube with 3 mL of 0.2 N sulfuric acid preservative solution. A backup of 4 g was also aliquoted, and all samples were stored at −20 °C until analysis.

Fecal score was assessed multiple times daily during the sampling week, graded on a scale of 1–7 using the Fecal Scoring System (Nestle Purina^®^ Petcare, St. Louis, MO, USA). Grade 1 represented dry, crumbly feces, while Grade 7 indicated liquid diarrhea.

Additionally, to determine the presence of the probiotic in the feces, full, fresh depositions of each animal were collected during the adaptation phase, on the third week, and on the last day of the sampling week of each period. In the first period, only feces from the sampling week were collected. Samples were stored at −20 °C until analysis.

Fasting blood samples were obtained at the end of each period. Blood was collected using non-heparinized tubes, which, after 40–60 min, were centrifuged at 1300× *g* for 10 min, and the serum was collected and frozen at −20 °C until analysis. Additional fresh blood samples were collected with EDTA tubes for CBC (Complete Blood Count) and determination of lymphocyte subsets. Additional aliquots were also utilized for the isolation of PBMCs (peripheral blood mononuclear cells) by Ficoll gradient centrifugation, and the cells were subsequently cryopreserved for further analysis of phagocytic activity.

### 2.3. Analytical Methods

After the sampling weeks, the feces collected for digestibility analysis were thawed, and dried in an oven at 60 °C until constant weight (3 days) to determine the dry matter content. Following the drying process, the feces collected from each animal during the 6-day collection period were ground, mixed, and a representative sample was taken for further chemical analysis.

The chemical analysis of diets and fecal samples followed the AOAC methods [32]. Feces and diets were analyzed for moisture (AOAC 930.15), ash (AOAC 942.05), crude protein (AOAC 954.01), crude fat (AOAC 954.02), and crude fiber (AOAC 962.09—2010). Gross energy was measured using an adiabatic calorimetry bomb. Total dietary fiber from the diets was analyzed following the AOAC 985.29 method.

Short-Chain Fatty Acids (SCFA) were analyzed by gas chromatography after an acid–base treatment of the samples, followed by extraction and derivatization with diethyl ether [33,34].

Urea, total cholesterol, haptoglobin, and glucose levels were determined in blood serum through photometric tests using an Olympus AU 400 Chemistry Analyzer (Beckman Coulter, Brea, CA, USA) with Olympus System Reagents^®^ (Olympus, Antrim, UK).

For the CBC, flow cytometry analysis was performed using a Sysmex™ XN-1000 (Kobe, Japan). 

Lymphocyte subset determination (CD4+ and CD8+) immunophenotyping was conducted with a BD FACSCanto™ II Flow Cytometer (BD and Company, Franklin Lakes, NJ, USA).

Phagocytic activity was measured by incubating leukocytes at 37 °C for 1 h with pHrodo™ Green *E. coli* BioParticles™ Conjugate (Thermo Fisher Scientific, Waltham, MA, USA). The cells were analyzed using a Cytoflex cytometer (Beckman Coulter, Brea, CA, USA) with CytExpert software v2.4 and a minimum of 15,000 cells were analyzed from each sample. Median Fluorescence Intensity (MFI) was determined on neutrophils, while total internalization was determined in monocytes and neutrophils. Total internalization refers to the total percentage of cells (100,000 cells culture per sample) that internalized pHrodo™ Green *E. coli* BioParticles™ Conjugate after the incubation.

Fecal ammonia was determined using the Ion Selective Electrode method [35].

Fecal IgA quantification was measured by ELISA sandwich using a Dog IgA ELISA Kit (Cat. No. E44-104, Bethyl Laboratories Inc., Montgomery, TX, USA). For calprotectin levels, the immunoturbidimetric Bühlmann fCAL^®^ turbo assay was used.

Probiotic fecal counts (CFU) were determined by culturing *B. velezensis* DSM 15544 following the methods described by the British Standards Institute [36]. 

DNA extraction of fresh feces was performed using the QIAamp Fast DNA Stool Mini Kit by Qiagen™ (Hilden, Germany). The quality of DNA extracts was evaluated using spectrophotometry (Nanodrop™, Wilmington, DE, USA)) and fluorimetry (Qubit™ HS, Wilmington, DE, USA).

Fecal microbiota taxonomy was determined by sequencing 16S rRNA V3-V4 regions using the Illumina^®^ MiSeq platform. Prior to sequencing, the 16S rRNA gene was amplified using universal primers [37] and a universal linker sequence together with indexes and sequencing primers by the Nextera XT Index kit (Illumina^®^, San Diego, CA, USA). Raw sequences, forward (R1) and reverse (R2), were imported into the QIIME2 platform [38]. Then, the DADA2 plugin [39] was used to denoise and filter the reads. The taxonomy of the resulting Amplicon Sequence Variants (ASV) was annotated using ‘blastn’ v2.2.29+ [40] against the 16S specific database from the NCBI (v. May 2022). Assigned taxonomies with an identity percentage lower than 97% were reassigned using the Naїve-Bayes algorithm [41] against SILVA v.138 SSU Ref NR 99 database. Data were normalized using the rarefaction technique from the ‘Phyloseq’ R package v1.50.0 [42] to perform alpha diversity analysis. Shannon, Simpson, and Richness indexes were calculated using the ‘vegan’ R package v4.0 [43].

To assess the potential impact of the experimental treatments on microbial taxonomy, the feature table was normalized using the ‘calcNormFactors’ function, with the trimmed mean of M-values (TMM) option. After normalization, the ‘limma’ [44] (version 3.42.2) function ‘voom’ was used to convert normalized counts to log2-counts-per-million and assign precision weights to each observation based on the mean-variance trend.

### 2.4. Calculations

The apparent digestibility of various nutritional components, including dry matter, organic matter, crude protein, ether extract, crude fiber, and gross energy, was determined using the formula:

(1) $$\mathrm{Apparent}\mathrm{Digestibility}=\frac{\mathrm{Ingested}-\mathrm{Excreted}}{\mathrm{Ingested}}\times 100$$

Metabolizable energy (ME) content of the diet was estimated using the experimental obtained values of digestible energy (DE) and digestible protein (DP) of the diet, following the FEDIAF Nutritional Guidelines formula [45]:

(2) $$\mathrm{ME}=\frac{\mathrm{DE}-(\mathrm{DP}\times 1.25)}{\mathrm{Food}\mathrm{Consumed}\;(g)}$$

### 2.5. Statistical Analysis

Statistical analyses for performance, fecal quality, alpha diversity, digestibility of nutrients, SCFA, and blood parameters were carried out using R software, version 4.0.5 for Windows. RStudio version 1.4.1106 was employed for data analysis, utilizing the analysis of variance (ANOVA) with the General Linear Mixed Model (GLMM) procedure and the ‘nlme’ package, version 3.1-153. Prior to analysis, normality and equal variances were assessed for all continuous variables categorized by dietary treatment using the Shapiro–Wilk test. The general model incorporated diet, period, animal group, and the interaction of diet and period as fixed effects, with subject as a random effect.

Additional orthogonal contrasts were conducted for fecal IgA results. These contrasts were performed using the R software’s LmerTest package, utilizing the lmer function to fit the mixed model. Initially, orthogonal contrasts were performed by comparing the CON group with the average of the two treatments, followed by comparing one treatment against the other. The contrasts followed this pattern: (−2, 1, 1) (0, −1, 1).

Regarding the microbiota analysis, after normalization, the ‘limma-voom’ R package v.48.3 [44] was employed, where the weighted linear regression model, including subject as a random effect, was fitted. Additionally, the Bray–Curtis dissimilarity matrix and Permutational Multivariate Analysis of Variance (PermANOVA) were conducted using the ‘vegan’ R package to determine possible changes in the ecosystem structure.

To study correlations between microbial abundances and SCFA fecal content parameters, the FeMaAslin2 [46] was used. The heatmap was constructed using ‘ComplexHeatmap’ (v.2.10.0) R package.

Results were presented as lsmeans ± standard deviation, and the significance level was set at α < 0.05 (α < 0.1 for trends).

## 3. Results

Throughout the study, the dogs remained in good health and showed no signs of compromised well-being. The only sampling incident was the inability to collect a fresh fecal sample during the third period. The average weight of the dogs remained stable across all diets, with no significant differences in body weight changes observed among diet groups. The mean weight change for the whole experiment was −0.1 ± 3.31 kg for the CON diet, +2.3 ± 3.68 kg for the SYN diet, and +0.4 ± 4.10 kg for the SYN+ diet. The daily energy intake, measured in kilocalories of metabolizable energy, was 1071 ± 123 for the CON diet, 1194 ± 159 for the SYN diet, and 1138 ± 174 for the SYN+ diet. These estimations were made based on the ME values calculated for the three experimental diets based on the FEDIAF formula.

In terms of probiotic survival along the gastrointestinal tract, plate counts of *Bacillus spp*. were recorded at 5.73 × 10^5^ ± 0.02 (SE) and 5.67 × 10^5^ ± 0.04 (SE) CFU/g feces for the SYN and SYN+ groups, respectively. In the CON group, counts were always below the minimum level of detection (less than 1000 CFU/g feces).

### 3.1. Fecal Quality

The results for the fecal consistency, dry matter content, and fecal mass output are shown in Table 2.

Fecal dry matter was lower in SYN and SYN+ treatments, without significant differences in the fecal score. SYN and SYN+ diets notably increased the daily fecal mass.

### 3.2. Apparent Digestibility of Nutrients and Metabolizable Energy

Table 3 presents the apparent fecal digestibility of the energy and macronutrients for each diet, alongside the calculated metabolizable energy content using the FEDIAF formula.

The apparent digestibility of dry matter and gross energy was found to be significantly lower in the SYN and SYN+ diets compared to the CON diet. Additionally, crude protein digestibility tended to be lower with the SYN diet. However, no differences were detected between diets in terms of crude fiber and fat digestibility. Although metabolizable energy was numerically similar between diets, the SYN diet exhibited significantly lower values, with a 2.6% decrease compared to the CON diet.

### 3.3. Fermentative Products in the Feces

The fecal concentration and molar ratio of short-chain fatty acids and ammonia is shown in Table 4.

Both experimental diets exhibited numerically higher concentrations of total SCFA compared to the CON diet, with the SYN diet reaching statistical significance. Specifically, acetate levels were elevated with the SYN diet, but not with the SYN+ diet. Branched-Chain Fatty Acids (BCFA) consistently demonstrated lower levels with both the SYN and SYN+ diets. In terms of the molar ratio, the SYN diet was associated with significantly higher percentages of acetate and lower percentages of propionate and butyrate. The percentage of butyrate was also lower in the SYN+ diet compared to the CON diet. Both test diets led to a decrease in the BCFA percentage. Fecal ammonia did not exhibit any significant differences between diets, although it numerically decreased with the SYN diet.

### 3.4. Fecal Microbiota

The sequencing of the 35 fecal samples yielded an average of 65,403 ± 9700 sequences per sample, resulting in a total of 165 ± 33 ASVs per sample. Assignments were made with a minimum similarity threshold of 97%.

**Figure 1:**
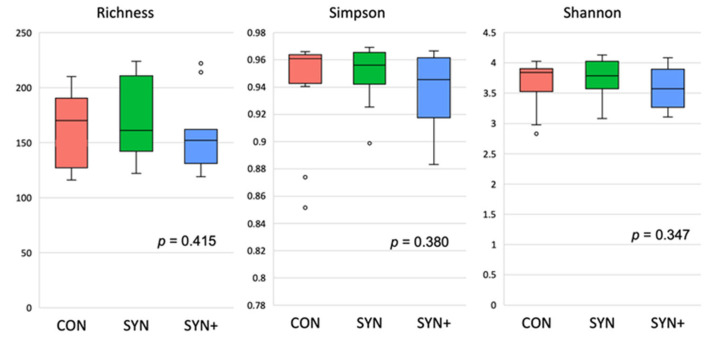
Alpha diversity of canine fecal microbiota at an ASV level from fecal samples and represented as Richness, Simpson, and Shannon indexes. CON: control diet low in fiber (in red); SYN: diet formulated with added fiber and supplemented with *B. velezensis* DSM 15544 (in green); SYN+: SYN diet with added spray-dried plasma (in blue). *p*-values for the diet effect were 0.415, 0.380, and 0.347 for Richness, Simpson, and Shannon indexes, respectively.

Alpha diversity indexes, including Richness, Simpson, and Shannon, were computed using ASVs and are depicted in Figure 1. No significant differences were observed among the various treatment groups, although the SYN+ diet appeared to exhibit lower alpha diversity values.

**Figure 2:**
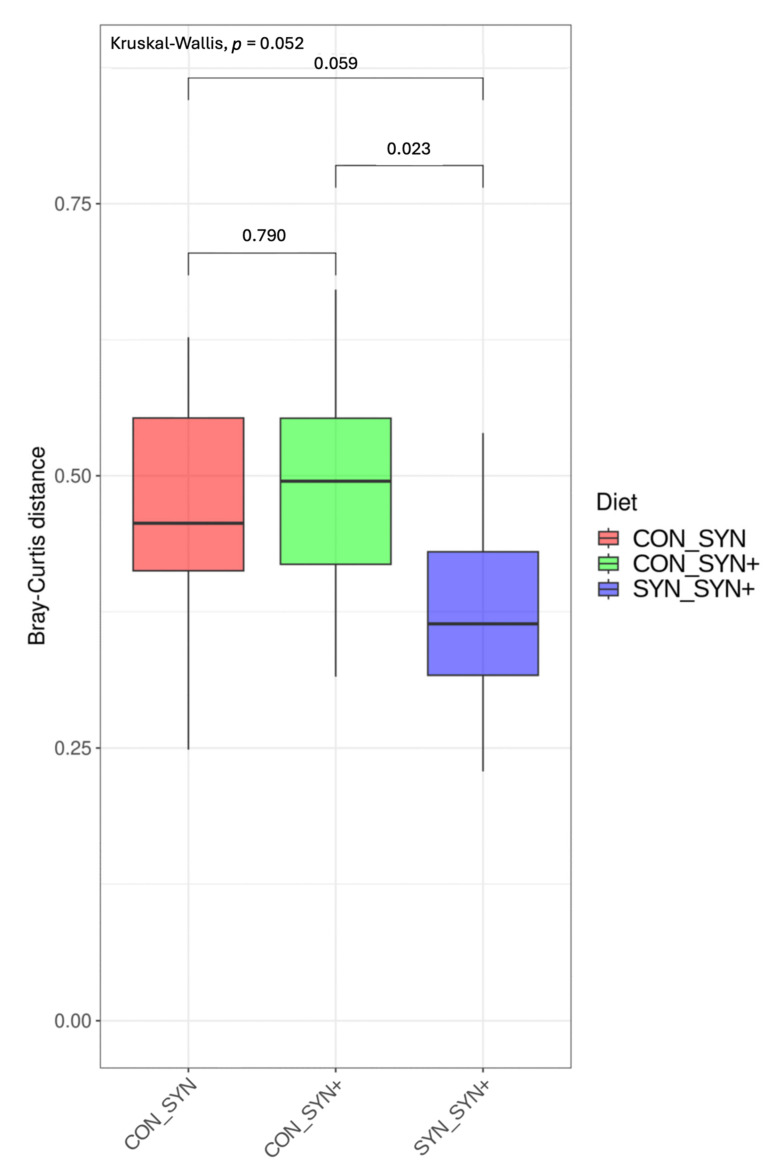
Boxplot of the Bray–Curtis distances of dog fecal microbiota samples at an ASV level. Bars represent the comparison between two different diets: the higher the distance, the more different diets are in terms of beta diversity. CON: control diet low in fiber; SYN: diet formulated with added fiber and supplemented with *B. velezensis* DSM 15544; SYN+: SYN diet with added spray-dried plasma.

Bray–Curtis distances at the ASV level were analyzed to assess the beta diversity of fecal microbiota samples. In the boxplot illustrating the comparisons between diets (Figure 2), the SYN (B) and SYN+ (C) diets exhibit greater proximity to each other in terms of Bray–Curtis distances compared to the CON diet (A). However, no significant differences or clustering were observed in the Principal Coordinates Analysis (PCoA) (Figure S1). Conversely, the PermANOVA analysis revealed a noteworthy contribution of diet, animal, and period to the variability of the samples (*p* < 0.001 for all three factors). Among these factors, animal presence was the most influential, accounting for 45.8% of the variability, while diet and period contributed 10.8% and 11.4%, respectively.

The relative abundances of different phyla were distributed into four main groups: *Bacteroidetes* (50.1 ± 7.18%), *Firmicutes* (23.0 ± 11.30%), *Fusobacteria* (14.7 ± 6.19%), and *Proteobacteria* (11.7 ± 5.17%). No significant differences were observed between diets. A graphical representation of phylum relative abundance distribution can be found in the Supplementary Material (Figure S2).

At the family level, the most abundant families were *Prevotellaceae* (30.2 ± 7.08%), *Bacteroidaceae* (16.26 ± 7.49%), *Fusobacteriaceae* (14.7 ± 6.19%), *Sutterellaceae* (7.9 ± 5.06%), and *Turicibacteraceae* (5.7 ± 8.51%). Some differences were found at the genus level when comparing diets. Higher levels of *Tyzzerella* were found with SYN and SYN+ compared to CON, while the abundance of *Catenisphaera* was lower. Additionally, genera such as *Megamonas*, *Howardella*, and *Phocaeicola* tended to be higher in the SYN+ diet compared to CON.

**Figure 3:**
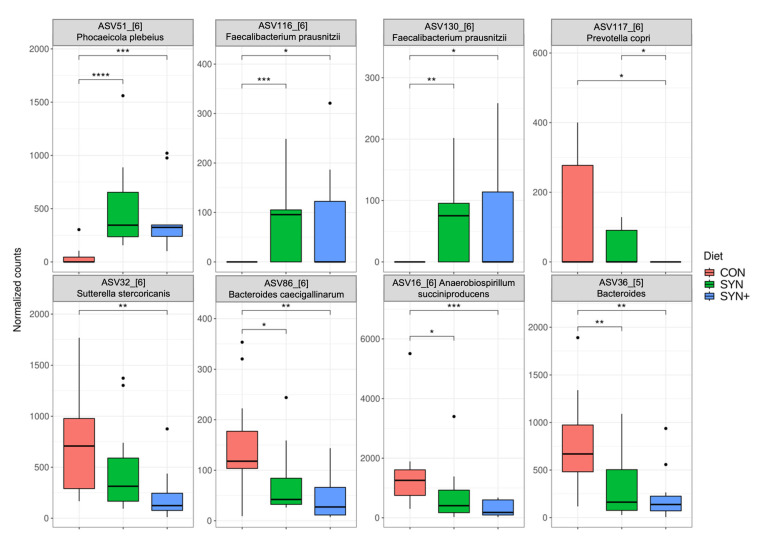
Boxplots of ASV normalized counts of fecal microbiota and comparisons between treatments. CON: control diet low in fiber; SYN: diet formulated with added fiber and supplemented with *B. velezensis* DSM 15544; SYN+: SYN diet with added spray-dried plasma. *: *p* < 0.05; **: *p* < 0.01; ***: *p* < 0.001; ****: *p* < 0.0001. The numbers within brackets are part of the ASV designation.

At the ASV level, there were also taxonomic differences between diets (Figure 3). The experimental diets SYN and SYN+ showed higher levels of *Phocaeicola plebeius* and *Faecalibacterium prausnitzii*, and lower levels of *Sutterella stercoricanis*, *Bacteroides caecigallinarum*, *Anaerobiospirillum succiniciproducens*, and *Bacteroides* ASV36-5. Conversely, the SYN+ diet had significantly lower counts of *Prevotella copri*.

**Figure 4:**
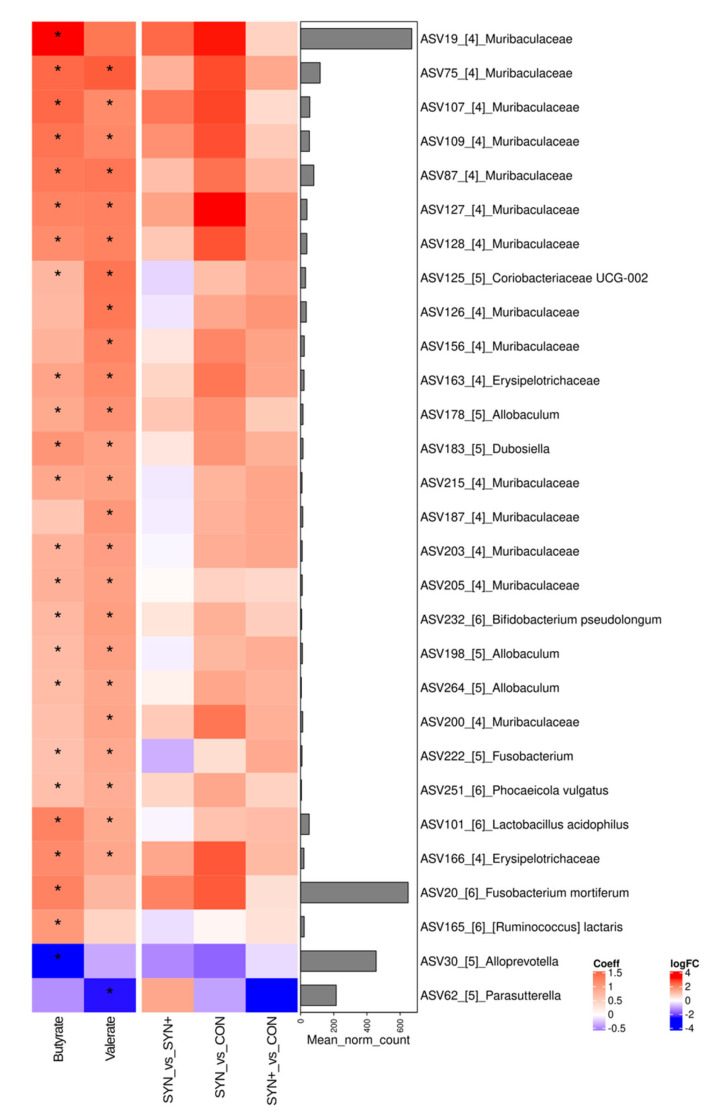
Heatmap of the correlation between ASV and specific SCFA on the left side, with additional diet and ASV correlations on the right side. Only SCFA that showed relevant correlations with more that 2 ASVs have been included in the graph. Red color on the left side means a positive correlation and blue means the opposite. On the right side, red indicates an overabundance of a specific ASV in the former diet in comparison to the other, while blue indicates an underabundance. The accompanying bar graph on the right displays the average normalized counts for each specific ASV. CON: control diet low in fiber; SYN: diet formulated with added fiber and supplemented with *B. velezensis* DSM 15544; SYN+: SYN diet with added spray-dried plasma. *: *p* < 0.05. The numbers within brackets are part of the ASV designation.

Finally, in Figure 4, correlations between ASV counts and specific SCFA concentrations are shown. To enhance the comprehension of the implications of each treatment, diet-ASV correlations are incorporated into the graph. A comparison between the SYN diet and CON reveals higher abundances of *Muribaculaceae* and *Erysipelotrichaceae* ASVs in SYN, significantly correlated with the increased production of butyrate and valerate. Similarly, SYN exhibits elevated levels of *Fusobacterium mortiferum*, significantly associated with heightened butyrate levels. SYN also showed a decreased abundance of a specific *Alloprevotella* ASV, negatively correlated with butyrate production. Likewise, compared to CON, SYN+ demonstrates a reduced abundance of a specific *Parasutterella* ASV, which is negatively correlated to valerate levels.

### 3.5. Serum Biochemistry and Immune Parameters

When examining serum biochemistry parameters, total cholesterol levels (mg/dL) exhibited significantly higher values (*p* = 0.004) in the SYN (234 ± 25.3 mg/dL) and SYN+ (231 ± 28.0 mg/dL) diets compared to the CON diet (218 ± 32.6 mg/dL). Additionally, serum urea levels (mg/dL) tended to be higher in the supplemented diets (*p* = 0.050), with mean values of 26.3 ± 4.74 for CON, 28.6 ± 7.76 for SYN, and 29.4 ± 8.36 for the SYN+ diet, respectively. However, other parameters such as glucose and serum electrolytes did not exhibit differences between diets.

Table 5 presents the complete blood cell counts (CBC) and lymphocyte subsets, including the leucocyte cell counts and phagocytic activity for each experimental group. No significant differences were observed in the counts of other blood cell types.

The SYN diet resulted in a significant increase in leucocytes, primarily attributed to elevated lymphocytes and neutrophils. However, there were no discernible differences in lymphocyte subsets due to the experimental treatments. The phagocytic activity of monocytes and neutrophils, as indicated by the percentage of internalization, and neutrophil Median Fluorescence Intensity (MFI) average values remained consistent across the diets. Nevertheless, MFI values exhibited a noteworthy progressive increase over the experimental periods (*p* < 0.001), with a suggestive trend (*p* = 0.074) for the interaction between the diet and period. Notably, animals on the CON diet during the initial period demonstrated the lowest numerical MFI value (8050), while those on this diet during the third period exhibited the highest (12,220) (Figure S3).

**Figure 5:**
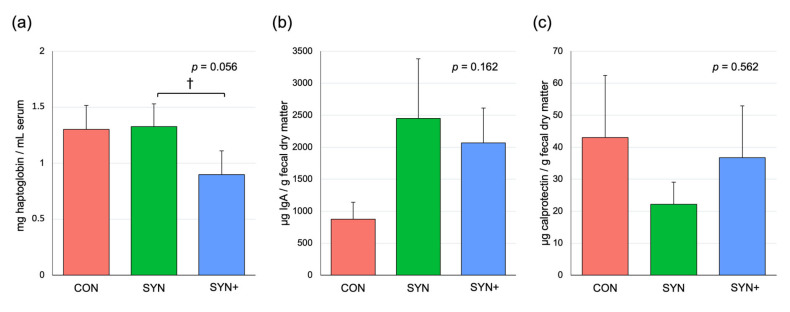
Bar graphs of average concentrations of serum haptoglobin (**a**), fecal secretory IgA (**b**), and fecal calprotectin (**c**) of dogs (n = 12) fed experimental diets. CON: control diet low in fiber (in red); SYN: diet formulated with added fiber and supplemented with *B. velezensis* DSM 15544 (in green); SYN+: SYN diet with added spray-dried plasma (in blue). †: *p* < 0.1. The error bars represent standard error of the mean.

Blood analysis also included serum haptoglobin, while fecal analysis included IgA and calprotectin too (Figure 5). Haptoglobin exhibited a trend (*p* = 0.056) toward lower concentrations with SYN+ compared to SYN. Fecal secretory IgA displayed numerically higher levels with SYN and SYN+ (*p* = 0.162), approaching statistical significance (*p* = 0.069) when both treatments were compared to CON. Fecal calprotectin remained unaffected by the dietary interventions.

## 4. Discussion

As stated before, the objective of this study was to investigate the impact of a synbiotic strategy in dogs, using different fibers and prebiotic sources combined with *B. velezensis* DSM 15544, and to assess if additional supplementation with SDP could enhance the expected benefits.

The probiotic *B. velezensis* DSM 15544 used in the study had demonstrated efficacy in various dog studies, showing beneficial effects on fecal odor and quality, microbiota communities, and nutrient digestibility [14,15,16,17]. Furthermore, different isolated prebiotics and prebiotic sources, including inulin, beet pulp, and pea fiber, were expected to complement the probiotic’s effects based on their potential to improve SCFA production and other positive outcomes in dogs [47,48]. The addition of SDP to this mix, we hypothesized, could further enhance the benefits of the synbiotic strategy through various mechanisms, such as providing passive immunity [49], improving intestinal integrity [26], and offering beneficial bioactive components [50].

### 4.1. Effects Driven by the Synbiotic Combination

Several consistent effects were seen with treatments including the synbiotic combination (SYN and SYN+ diets), suggesting that these effects were mainly driven by the inclusion of different prebiotic sources combined with the *Bacillus* strain probiotic.

Fecal quality results revealed an increase in fecal mass with the supplemented diets, accompanied by a decrease in fecal dry matter, which reached significance only in the SYN+ diet. These findings align with the known water-holding and bulking effects of fibers [51]. Notably, fecal consistency scores remained unaffected by the diet, indicating that the external fecal appearance did not change. The probiotic may have contributed to maintaining the fecal scores [17], although other studies [52] have demonstrated that fiber-enriched diets can reduce the fecal dry matter content without altering the consistency.

Supplemented diets led to a decrease in the dry matter digestibility and a significantly lower level of metabolizable energy. This could be attributed to their higher fiber levels, which can impact the transit time and the enzymatic digestion of macronutrients, thereby affecting the digestibility [53,54]. Additionally, fibers generally provide reduced energy to dogs as they are not efficiently fermented compared to other species. Regarding the digestibility of nutrients, the influence of the probiotic was expected to be small, as indicated in other studies [14,15].

One unexpected effect observed with supplemented diets was an increase in the total cholesterol. Typically, elevated levels of fiber and prebiotics are associated with a decrease in cholesterol levels [55]. A similar phenomenon occurs in humans supplemented with probiotics, although the outcome can vary depending on the specific strain and dosage [56]. In dogs, a study identified a regulatory effect of a probiotic (*Enterococcus faecium* EE3) on cholesterol levels. This probiotic supplementation promoted physiological levels by adjusting cholesterol concentrations in dogs with low or high blood cholesterol levels [57]. However, another study utilizing *B. velezensis* DSM 15544 observed no significant difference in cholesterol levels in dogs. Furthermore, there was a numerical decrease in cholesterol values after 30 days, with lower baseline cholesterol levels recorded [15]. Consequently, the underlying cause or mechanism of this cholesterol increase remains unidentified.

Urea levels exhibited a tendency to increase in the supplemented diets, while apparent protein digestibility tended to be slightly lower, with protein levels remaining relatively consistent across all diets. The fermentation of fibers, as indicated by the increase in SCFA, might have contributed to the proliferation of bacterial mass in the gut and subsequent higher microbial nitrogen excretion in feces. This could suggest that real protein digestibility values for fiber diets could be higher, resulting in greater protein absorption and urea generation. In fact, beyond the impact of fiber, the addition of *B. velezensis* DSM 15544 could have also contributed to higher protein assimilation, as proven by the increased protein digestibility observed in a canine study [15].

Synbiotic supplemented diets also showed a consistent impact on saccharolytic fermentative activity, as assessed by an increase in SCFA and a decrease in BCFA, attributed to the fermentability of the dietary fiber present in both supplemented treatments. *B. velezensis* could further contribute to the SCFA increase, given its acetate-producing properties [58], as evidenced in certain dog studies [14,17]. With the SYN diet, increases were observed in both the concentration and molar percentage of acetate. Acetate has been found to have numerous extragastrointestinal benefits, such as decreasing lipid accumulation, reducing blood pressure, and improving cognitive function [59].

Regarding possible changes promoted by the synbiotic strategy on the gut ecosystem, the 16S rRNA gene analysis revealed no major differences in the alpha diversity. However, smaller Bray–Curtis distances between the SYN and SYN+ diets (compared to CON) indicated similar ecosystems within the supplemented groups. These results suggest that the synbiotic strategy could have led to changes in the structure of the microbiota despite no changes in the alpha diversity.

A taxonomical analysis revealed that major phyla were consistent with those previously described for dogs, albeit *Firmicutes* and *Fusobacteria* typically represent a higher proportion of microbial reads in other studies [60,61,62,63]. Regarding changes induced by diets, both supplemented diets exhibited consistent changes in particular microbial groups. With these two diets, higher levels of *F. prausnitzii*, a beneficial bacterial species in humans [64] that has also been described to increase in dogs fed a high-fiber diet [65], were recorded. *P. plebeius* was also higher in the supplemented diet groups. *P. plebeius* is a bacterium that has been negatively correlated with type 2 diabetes in humans [66]. Its genus, previously classified as *Bacteroides*, has been associated with high-fiber diets in dogs and SCFA production [67,68]. However, changes were also seen in other bacteria not traditionally considered beneficial. Higher counts were found for the *Tyzzerella* genus, which has been reported as a possible negative biomarker in humans for heart disease [69] and linked with inflammation and ectopic fat [70]. However, in dogs, lower abundances of this genus have been observed in dogs with diarrhea [71].

### 4.2. Specific Effects Associated with SDP Supplementation

As stated before, the SYN+ diet aimed to assess if SDP could provide additional benefits to the synbiotic strategy, mainly in terms of intestinal health and/or the immune response of the animals. In general, specific changes attributed to SDP supplementation were scarce; however, some insights suggest that it could have played a role.

Different studies have demonstrated distinctive prebiotic actions of SDP on the intestinal microbiota of different animal species. A study using a mice model, at an 8% inclusion of SDP, evidenced significant increases in different bacterial groups associated with gut health and mucosal barrier restoration [72]. Other studies in pigs, with lower SDP dosages, also demonstrated significant effects, with increased *Lactobacilli* and *Ruminococcus* levels at 1% [73] and 5% [74] inclusion rates. Notably, none reported alpha diversity shifts with SDP supplementation, but [74] observed clear beta diversity clustering in the supplemented group. In the present study, we were not able to find such consistent effects on taxonomy or diversity with SDP; however, the levels of supplementation (0.4% in the present study) were not as high as in previous studies. Despite that, some effects were seen specifically with the SYN+ diets.

Regarding the fermentative activity, increases in the fecal concentration of SCFA were not as marked with the SYN+ diet as with SYN, and differences between both diets were found in terms of the molar ratio of some SCFA. These differences could be related to changes in particular microbial groups. For example, the SYN diet seemed to have higher counts of ASVs significantly correlated with SCFA production, such as *Muribaculaceae*, *Erysipelotrichaceae*, or *F. mortiferum*, and negatively correlated with butyrate production like *Alloprevotella*, when compared to CON. This differential impact could be behind the differences found between these two diets regarding their fermentation profile, with lower molar ratios of acetate and higher molar ratios of propionate registered with the SYN+ diet. Moreover, the SYN+ diet exerted distinctive effects on other species, including increased *Megamonas*, a SCFA producer associated with weight gain [75] and prebiotic consumption in dogs [66,76], decreased *P. copri*, linked to rheumatoid arthritis in humans [77] and an increased body condition score in dogs [78], and increased *Phocaeicola*, whose positive effects have previously been noted.

Regarding the potential impact of SDP supplementation on the immune response, it is intriguing that increased levels of circulating blood leukocytes, particularly lymphocytes and neutrophils, were only observed in the SYN diet, but not in the SYN+ diet. Previous research on rabbits demonstrated that *B. velezensis* can elevate proinflammatory cytokines such as IL-1β and IL-8, thereby promoting the recruitment of leukocytes [79]. This increase could, therefore, be seen as a result of the probiotic supplementation. The lack of effect with the SYN+ diet could be due to some interaction between the probiotic and SDP. In this regard, SDP has been described to possess anti-inflammatory properties in pigs [80], possibly by dampening inflammatory responses and enhancing the expression of IL-10 and TGF-β, as demonstrated in a mouse model of inflammation [81]. Other studies in mice led to similar conclusions, reducing gut mucosal inflammation in a model of IBD [82] and neuroinflammation in a senescence model [83]. Consequently, SDP might have attenuated the activation and proliferation of neutrophils and lymphocytes. Consistent with these findings is the numerical reduction in serum haptoglobin, an inflammatory acute-phase protein, observed only with the SDP-containing diet.

## 5. Conclusions

The dietary synbiotic strategy assessed in this study, which combined different sources of fibers, prebiotics, and the probiotic strain *B. velezensis* DSM15544, exhibited a positive influence on the intestinal health of dogs. This strategy enhanced microbial fermentative activity without adversely affecting the fecal quality and only marginally reducing the digestibility of dry matter and energy. Moreover, it demonstrated favorable effects on both immunity, with increases in fecal IgA, and the gut microbiota, with increases in certain beneficial species. The additional supplementation of SDP provided some insights into a distinct impact on the microbiota and immune response, although specific effects attributed to SDP were generally scarce.

## Figures and Tables

**Table 1:** Analyzed chemical composition of experimental diets (% as-fed).

	CON	SYN	SYN+
Dry matter (%)	92.96	93.40	93.55
Crude protein (%)	27.40	27.30	27.70
Crude fat (%)	15.15	15.04	15.82
Ashes (%)	6.33	6.47	6.38
Crude fiber (%)	1.84	3.65	3.65
Total dietary fiber (%)	5.25	7.90	8.70

Note: CON: control diet low in fiber; SYN: diet formulated with added fiber and supplemented with *B. velezensis* DSM 15544; SYN+: SYN diet with added spray-dried plasma.

**Table 2:** Fecal characteristics of dogs (*n* = 12) fed control or experimental diets (mean ± SD).

	CON	SYN	SYN+	*p* Value
Fecal score	2.85 ± 0.433	2.80 ± 0.223	2.81 ± 0.478	0.893
Fecal dry matter (%)	35.3 ^a^ ± 2.71	32.4 ^ab^ ± 5.05	32.0 ^b^ ± 3.02	0.040
Fecal mass (g FM/day)	86 ^a^ ± 15.2	136 ^b^ ± 23.4	126 ^b^ ± 32.3	<0.001

Note: CON: control diet low in fiber; SYN: diet formulated with added fiber and supplemented with *B. velezensis* DSM 15544; SYN+: SYN diet with added spray-dried plasma. All were extruded diets. FM: fresh matter. Means within a row with different superscripts showed statistical differences (*p* < 0.05).

**Table 3:** Apparent total tract fecal digestibility and metabolizable energy content (mean ± SD) of the experimental diets tested in dogs (*n* = 12).

	CON	SYN	SYN+	*p* Value
Dry matter (%)	86.9 ^a^ ± 1.60	84.4 ^b^ ± 2.31	85.0 ^b^ ± 2.94	<0.001
Crude protein (%)	86.4 ± 1.90	85.2 ± 2.00	85.7 ± 2.72	0.071
Crude fat (%)	96.2 ± 0.51	96.1 ± 0.59	96.3 ± 0.68	0.360
Crude fiber (%)	54.1 ± 8.99	50.3 ± 8.93	54.7 ± 9.53	0.262
Energy (%)	90.6 ^a^ ± 1.23	88.2 ^b^ ± 1.66	88.7 ^b^ ± 2.20	<0.001
Metabolizable energy (kcal/kg as fed), FEDIAF	4045 ^a^ ± 53.0	3940 ^b^ ± 73.1	4008 ^a^ ± 97.3	<0.001

Note: CON: control diet low in fiber; SYN: diet formulated with added fiber and supplemented with *B. velezensis* DSM 15544; SYN+: SYN diet with added spray-dried plasma. All were extruded diets. FEDIAF: Fédération Européenne de l’Industrie des Aliments pour Animaux Familiers. Means within a row with different superscripts showed statistical differences (*p* < 0.05).

**Table 4:** Fermentative products in fecal samples of dogs (*n* = 12), including SCFA, ammonia, and SCFA molar ratio. Data presented as mean and its standard deviation.

Concentration (µmol/g)	CON	SYN	SYN+	*p* Value
Total SCFA	176 ^a^ ± 23.5	209 ^b^ ± 46.5	195 ^ab^ ± 21.5	0.018
Acetate	94 ^a^ ± 12.1	116 ^b^ ± 23.4	104 ^ab^ ± 13.2	0.004
Propionate	58.0 ± 11.01	67.3 ± 19.87	66.5 ± 10.26	0.066
Butyrate	16.8 ± 2.44	19.4 ± 7.59	18.5 ± 6.96	0.372
Valerate	0.40 ± 0.078	0.62 ± 0.268	0.83 ± 1.011	0.189
Total BCFA	7.34 ^a^ ± 1.063	5.63 ^b^ ± 1.626	5.42 ^b^ ± 0.598	0.001
Ammonia (mg/g)	20.8 ± 10.37	12.3 ± 9.70	22.6 ± 19.54	0.133
Molar Ratio (%SCFA)				
Acetate	53.2 ^a^ ± 2.36	55.9 ^b^ ± 2.63	53.2 ^a^ ± 3.20	0.015
Propionate	32.8 ^ab^ ± 2.73	31.7 ^a^ ± 3.47	34.0 ^b^ ± 2.54	0.026
Butyrate	2.46 ^a^ ± 0.387	1.79 ^b^ ± 0.446	1.80 ^b^ ± 0.258	0.001
Valerate	0.45 ± 0.043	0.53 ± 0.154	0.65 ± 0.498	0.277
Total BCFA	4.22 ^a^ ± 0.724	2.76 ^b^ ± 0.749	2.80 ^b^ ± 0.359	<0.001

Note: CON: control diet low in fiber; SYN: diet formulated with added fiber and supplemented with *B. velezensis* DSM 15544; SYN+: SYN diet with added spray-dried plasma. All were extruded diets. SCFA: Short-Chain Fatty Acids; BCFA: Branched-Chain Fatty Acids. Means within a row with different superscripts showed statistical differences (*p* < 0.05).

**Table 5:** Blood leucocyte counts, blood lymphocyte subsets counts, and phagocytic activity of blood leukocytes in dogs (*n* = 12) fed the experimental diets. Data presented as mean ± SD.

	CON	SYN	SYN+	*p* Value
Leucocytes (count^3^/μL)	7.01 ^a^ ± 1.243	7.99 ^b^ ± 1.529	7.03 ^a^ ± 1.023	0.015
Lymphocytes (count^3^/μL)	2.12 ^a^ ± 0.478	2.32 ^b^ ± 0.628	2.02 ^a^ ± 0.469	0.003
Monocytes (count^3^/μL)	0.46 ± 0.085	0.50 ± 0.161	0.45 ± 0.110	0.313
Neutrophils (count^3^/μL)	4.16 ^a^ ± 0.971	4.91 ^b^ ± 1.487	4.31 ^ab^ ± 0.699	0.049
CD8 (count^3^/μL)	0.54 ± 0.227	0.56 ± 0.269	0.52 ± 0.237	0.489
CD4 (count^3^/μL)	0.62 ± 0.159	0.63 ± 0.155	0.59 ± 0.129	0.707
Total internalization (%)	95.3 ± 1.92	95.3± 1.78	95.1 ± 1.90	0.906
Neutrophil MFI *	10,257 ± 2143	10,584 ± 1803	10,455 ± 2120	0.713

Note: CON: control diet low in fiber; SYN: diet formulated with added fiber and supplemented with *B. velezensis* DSM 15544; SYN+: SYN diet with added spray-dried plasma. All were extruded diets. MFI: Median Fluorescence Intensity. * Arbitrary units. Means within a row with different superscripts showed statistical differences (*p* < 0.05).

## Data Availability

The data presented in this study are available on request from the corresponding author. The data are not publicly available due to privacy restrictions.

## Supplementary Materials

The following supporting information can be downloaded at: https://www.mdpi.com/article/10.3390/ani14233366/s1, Figure S1: Bray-Curtis principal coordinate analysis plot; Figure S2: Phylum relative abundance of each animal in each treatment, period, and group; Figure S3: Average Median Fluorescence Intensity values from each period of the study.
