# Supplementary material for: Use of Different Synbiotic Strategies to Improve Gut Health in Dogs
Source: Animals (Basel). 2024 Nov 22;14(23):3366. doi: 10.3390/ani14233366 (PMC11640470; doi:10.3390/ani14233366)
Supplement: Supplementary file 1 [file animals-14-03366-s001.zip › animals-3286170-supplementary.pdf]

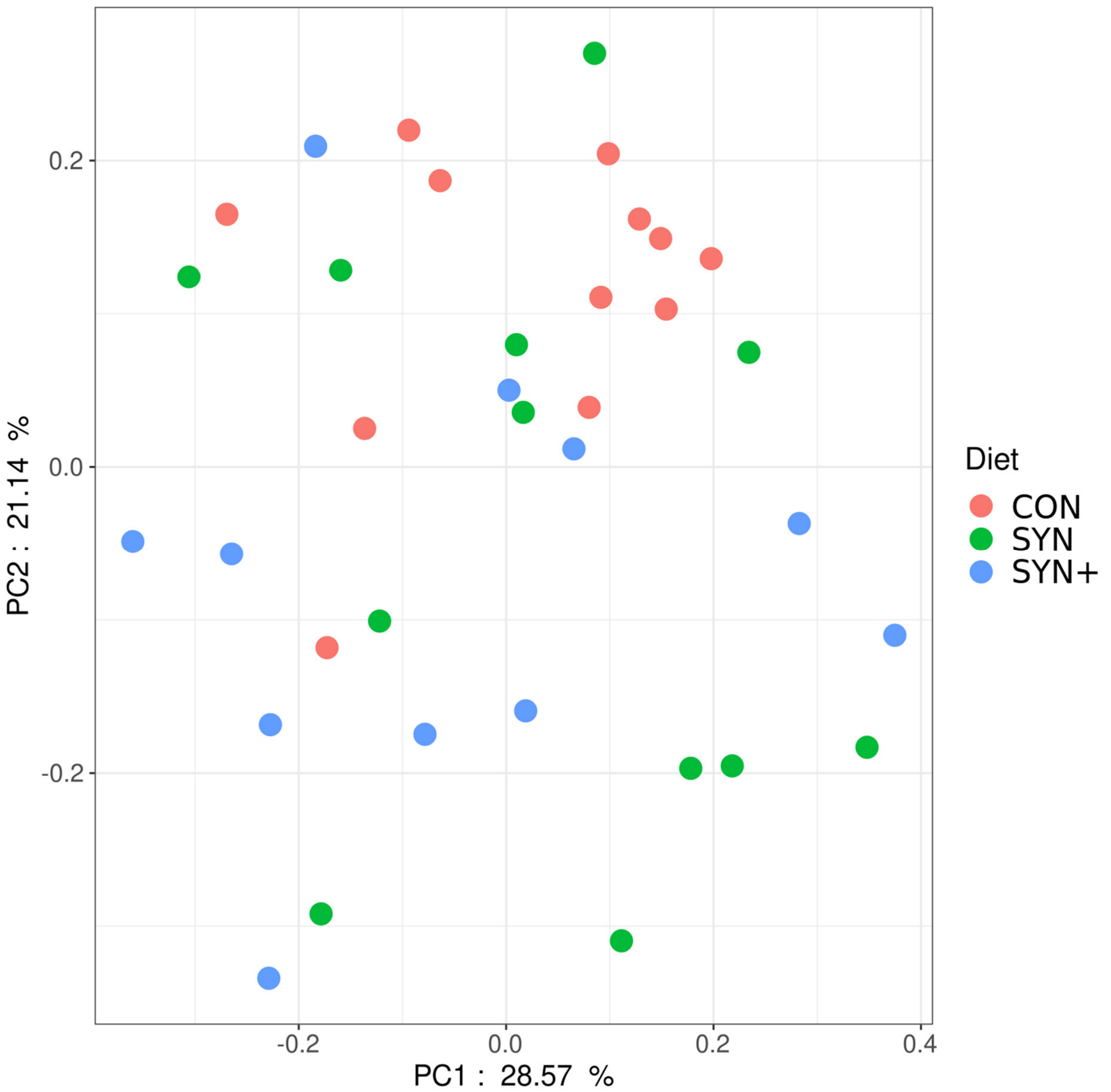

Figure S1. Bray-Curtis principal coordinate analysis plot of sequencing data from dog's fecal microbiota (*n* = 12). CON: control diet low in fiber; SYN: diet formulated with added fiber and supplemented with *B. velezensis* DSM 15544; SYN+: SYN diet with added spray-dried plasma.

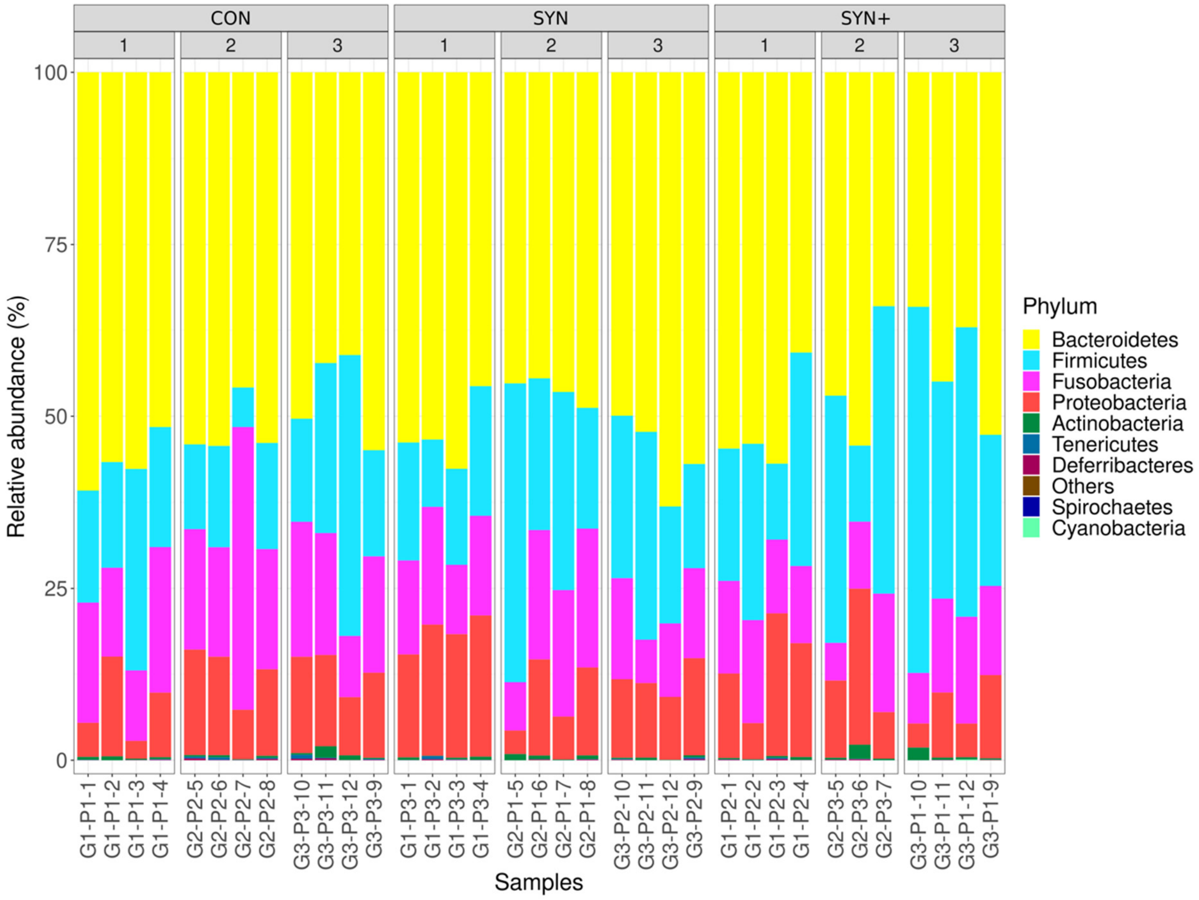

Figure S2. Phylum relative abundance of each animal in each treatment, period, and group. CON: control diet low in fiber; SYN: diet formulated with added fiber and supplemented with *B. velezensis* DSM 15544; SYN+: SYN diet with added spray-dried plasma. The x-axis represents individual animal samples, where 'G' indicates the group to which the animal belonged, 'P' represents the collection period, and the final number is the dog identifier.

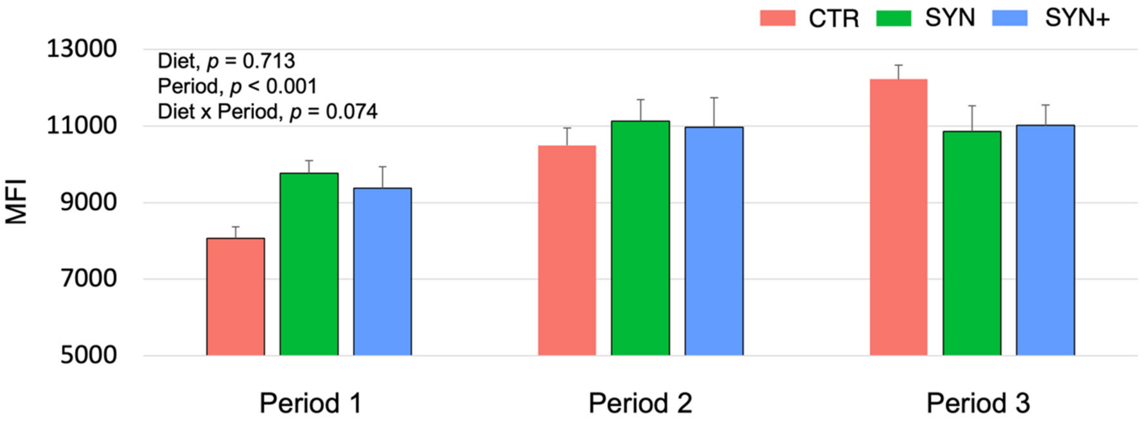

Figure S3. Average Median Fluorescence Intensity (MFI) values, expressed in arbitrary units, from each period of the study. CON: control diet low in fiber; SYN: diet formulated with added fiber and supplemented with *B. velezensis* DSM 15544; SYN+: SYN diet with added spray-dried plasma.
